# Fixed lateral unicompartmental knee replacement is a reliable treatment for lateral compartment osteoarthritis after mobile-bearing medial unicompartmental replacement

**DOI:** 10.1007/s00167-023-07573-y

**Published:** 2023-09-28

**Authors:** Joseph T. Lazzara, Lachlan W. Arthur, Cathy Jenkins, Christopher A. F. Dodd, Stephen J. Mellon, David W. Murray

**Affiliations:** 1 Nuffield Department of Orthopaedics, Rheumatology and Musculoskeletal Sciences (NDORMS), University of Oxford, Oxford, UK; 2 Nuffield Orthopaedic Centre, Oxford University Hospitals NHS Foundation Trust, Oxford, UK

**Keywords:** Bi-unicompartmental knee replacement, Revision unicompartmental knee replacement, Oxford unicompartmental knee replacement, Fixed lateral oxford

## Abstract

**Purpose:**

Lateral osteoarthritis following medial unicompartmental knee replacement (UKR) is usually treated with total knee replacement, however, lateral UKR is a less invasive option that preserves a well-functioning medial UKR. This study aimed to determine the 5-year outcome of the cemented Fixed Lateral Oxford UKR (FLO) when used for the treatment of severe lateral disease after medial Oxford unicompartmental knee replacement.

**Methods:**

Forty-four knees with lateral bone-on-bone osteoarthritis (*n = *43) and avascular necrosis (*n = *1) treated with the FLO following medial Oxford UKR were followed up prospectively. The Oxford Knee Score (OKS) and Tegner Activity Score (TAS) were collected pre- and post-operatively. Life-table analysis was used to determine survival rates.

**Results:**

The mean patient age at the time of FLO surgery was 74.4 years with a mean time of 12.1 years between the primary medial UKR and the conversion to a bi-UKR with a FLO. Mean follow-up of the FLO was 3.5 years. After FLO no intra-operative or medical complications, re-admissions, or mortality occurred. There was one reoperation in which a bearing was exchanged for a medial bearing dislocation. There were no revisions of the FLO, so the FLO survival rate at 5 years was 100% (24 at risk). The mean pre-operative OKS was 22, which significantly (*p < *0.0001) improved to a mean of 42, 42, and 40 at 1, 2, and 5 years, respectively. The median TAS had a non-significant improvement from 2.5 (Range 0–8) pre-operatively to 2 (Range 1–6) at 5 years postoperatively.

**Conclusion:**

The FLO is a reliable treatment for lateral osteoarthritis following medial UKR. At 5 years there was a 100% survival of the FLO with a mean OKS of 40.

**Level of evidence:**

IV, Prospective Case Series.

## Introduction

Medial compartment osteoarthritis of the knee can be treated with either Unicompartmental Knee Replacement (UKR) or Total Knee Replacement (TKR). The National Joint Registry suggests that UKR accounts for 9% of all primary knee operations in the United Kingdom and that the proportion is increasing [2]. When compared to TKR, medial UKR has been shown to have a faster recovery with fewer medical complications, better clinical outcomes, with better range of motion, function, and satisfaction. [3, 8, 15, 24, 26].

The medial Oxford UKR (OUKR) is the most widely used UKR and lateral compartment osteoarthritis is the most common reason for its revision, with TKR being the most common treatment [17]. However, a lateral UKR, with retention of a well-functioning medial UKR, is an alternative treatment [20, 25]. This operation exposes the patient to less intraoperative risk than TKR [13]. It is referred to as a staged bi-compartmental UKR (bi-UKR). Results of staged bi-UKRs, using predominantly mobile bearing UKR, were reported by Pandit et al. and showed significant improvement in patient functional scores when compared to their pre-operative status [20]. Further, a recent systematic review concluded both simultaneous and staged bi-UKR were feasible and viable surgical options for the treatment of bi-compartmental femorotibial osteoarthritis [25].

The 20-year survival of the medial OUKR is approximately 90% with the most common reason for revision being lateral osteoarthritis [22]. The mobile bearing lateral OUKR has not performed as well as the medial due to bearing dislocation because the lateral ligaments are lax in flexion [7, 19, 23]. The initial design had a flat tibial component. This was superseded with a domed tibial component which more accurately restored normal anatomy and kinematics [6]. Although the Domed Lateral OUKR (DLO) had a lower dislocation rate than the flat lateral, the dislocation rate was still higher than that of the medial OUKR [11]. To address this issue the Fixed Lateral OUKR (FLO) was introduced. The FLO is interchangeable with the DLO so if a surgeon is concerned about dislocation during a DLO procedure they can implant a FLO instead. Alternatively, the FLO can be used for primary replacement of the lateral compartment [1]. It is now routinely used in the elderly and for staged bi-UKR when the advantages of lower wear and improved kinematics are less important.

There is currently no published clinical data available for the use of the FLO implant in staged bi-UKR. Therefore, this paper aims to report the 5-year results of a series of patients with a medial mobile-bearing UKR who subsequently developed severe lateral disease and were treated with an FLO as a staged bi-UKR procedure. It is hypothesised that patients with lateral osteoarthritis revised with a FLO will have a clinical outcome that is as good, if not better, than results reported in the literature for patients revised with a TKR.

## Materials and methods

**Fig. 1:**
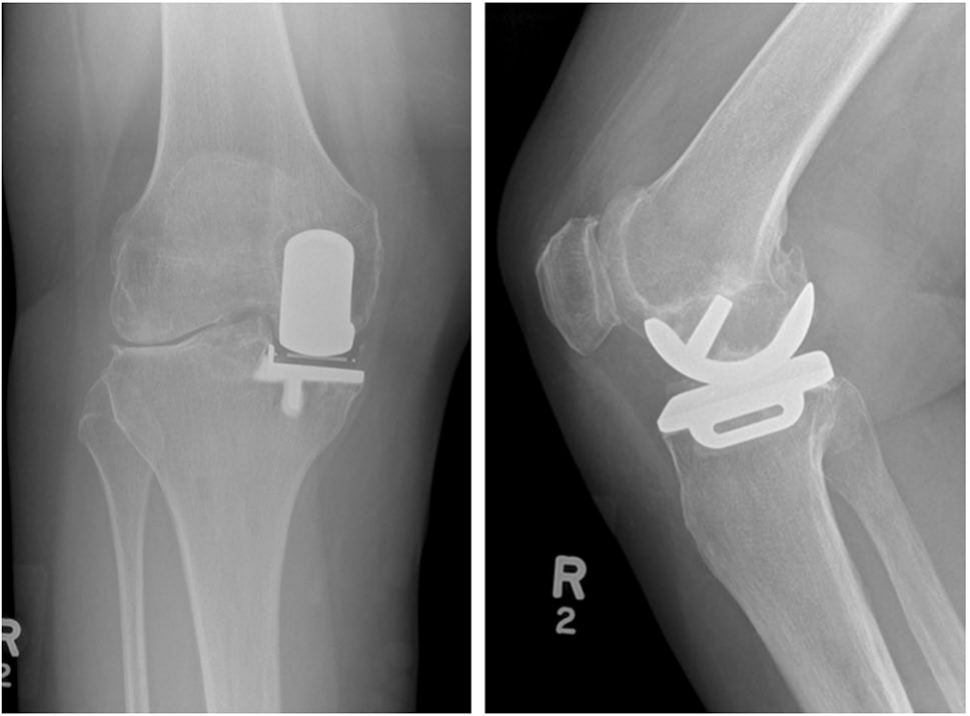
AP-X-ray (Left) and Sagittal-X-ray (Right) of medial OUKR with lateral osteoarthritis progression prior to conversion to bi-UKR

**Fig. 2:**
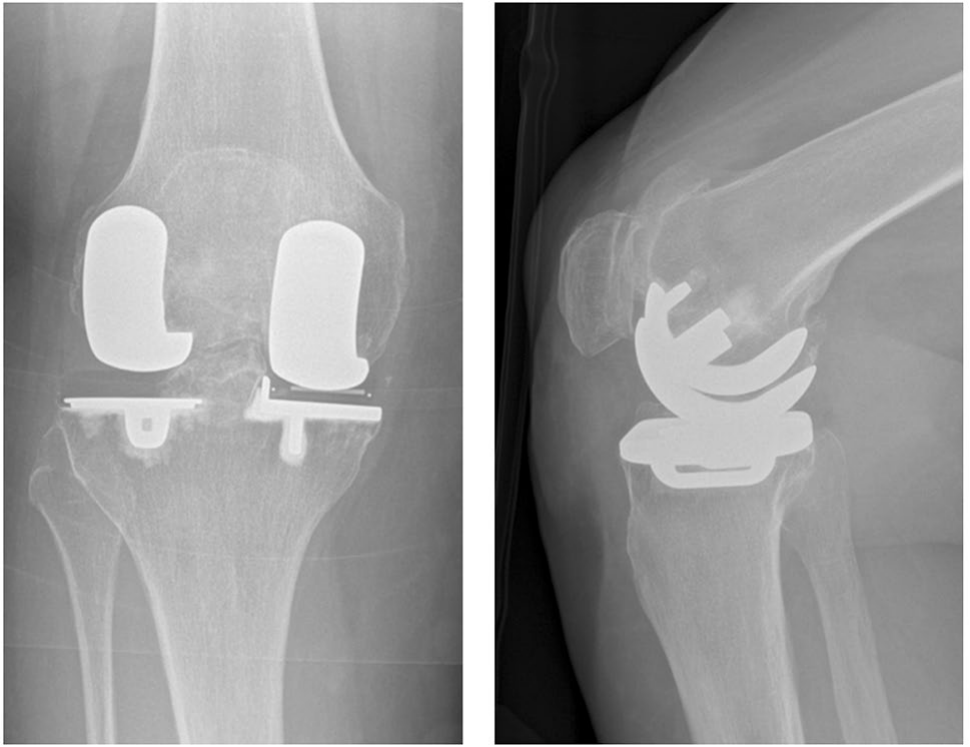
Postoperative AP-Xray (Left) and Sagittal-Xray (Right) of Fixed Lateral Oxford bi-unicompartmental knee replacement with well-aligned components (5-year OKS of 45 at last follow-up)

Between 2015 and 2022, 44 knees in 44 patients who had lateral disease progression following medial OUKR were treated with the FLO by two surgeons (Table 1). The indications for the staged bi-UKR procedure with an FLO were severe symptoms, lateral bone-on-bone osteoarthritis (*n = *43) or avascular necrosis (AVN, *n = *1) and a well-functioning medial UKR. The state of the patellofemoral joint (PFJ) was not considered to be a contra-indication with many having exposed bone in the PFJ. Four knees (9%) had full-thickness cartilage loss on the lateral patellar facet, 2 (4.5%) on the medial patellar facet, and 4 (9%) on the femoral trochlea. Valgus deformities were corrected so the procedure restored the pre-disease leg alignment. The staged bi-UKR procedure with the FLO was performed using the same technique as a cemented primary FLO [27], except that the original medial parapatellar skin incision is opened and extended allowing the lateral compartment to be entered in the normal way with a lateral parapatellar approach. The vertical tibial cut is made through the patella tendon, so it is approximately parallel to the medial vertical cut. Care is taken to avoid the avulsion of the tibial eminence. The components are positioned anatomically so that normal ligament tension is restored with the lateral ligaments being tight in extension and lax in flexion. If a medial bearing exchange is required, this is done through the old medial parapatellar approach. Pre-operative radiographs showing medial mobile-bearing OKRs are shown in Fig. 1, and post-operative radiographs demonstrating bi-unicompartmental knee replacement with FLO in Fig. 2.

**Table 1:** Patient Demographics for Patients who had a staged bi-UKR to revise a medial Oxford Unicompartmental Knee Replacement with lateral disease progression with the Fixed Lateral Oxford (FLO)

Demographic category	Value mean (SD)
Sex (female/male)	26/18
Age at operation (years)-primary medial OUKR	62.3 (9.1)
Time to revision for lateral disease progression (Years)	12.1 (3.8)
Age at operation-revision with FLO	74.4 (8.4)
Body mass index	28.7 (5.9)

This is a retrospective analysis of prospectively collected data. Clinical assessments were performed by an independent physiotherapist before and after the FLO and included the Oxford Knee Score (OKS) and Tegner Activity Score (TAS). Outcome scores were collected at 1, 2, 5, and 7 years post-operatively. Information about complications re-operations and revisions was collected and the revision/re-operation status of all knees was known.

### Ethical approval

Ethical approval was sought from the local research ethics committee chair (Oxfordshire Ethics Committee C) with formal approval deemed unnecessary under the National Health Service research governance arrangements.

### Statistical analysis

Statistical analysis for this study was performed in Microsoft Excel and IBM Statistical Package for the Social Sciences (SPSS) software. Paired *t*-tests were used to compare pre- and post-operative clinical scores at various time points. Linear regression analysis was used to investigate correlations between clinical data and other variables including age at the time of lateral UKR operation, extent of cartilage/bone loss for the involved femur or tibia, gender, body mass index (BMI), and time between medial and lateral UKR operations. Survival rates were calculated using the life table method, with failure defined as any re-operation, any re-operation related to the FLO and conversion to TKR. The primary outcome measure was post-operative OKS. A *p* value of < 0.05 was considered statistically significant.

## Results

There were 44 patients with a medial OUKR who were revised with a bi-UKR using an FLO for lateral disease progression. The demographics of these patients are summarised in Table 1. There were no intraoperative complications, medical complications, re-admissions, and no early mortality for any of the lateral UKR procedures. No patients were lost to follow-up. There was one re-operation for a medial bearing dislocation, which occurred 1.4 years after the FLO and was successfully treated by an insertion of a new bearing. The dislocation occurred 14.2 years after the initial medial UKR. There were no re-operations related to the FLO, no revisions of the FLO, and no conversions to a TKR. 2 patients died of unrelated causes when both knees were intact and in good condition, with their last scores taken at 2 years.

In the survival analysis (Table 2) with all re-operations considered to be a failure the survival rate was 97% at 5 years. The 5-year survival for re-operation or revision related to the FLO was 100%. The 5-year survival for revision to TKR was 100%.

**Table 2:** Life table analysis of bi-UKR study group

Post-operative year	Number of bi-UKRs	Failures	Number of bi-UKRs at risk	Cumulative survival (%)	95% CI
1	44	0	41	100	100–100%
2	38	1	32	97	91–100%
5	24	0	14	97	88–100%
7	3	0	2	97	69–100%

**Fig. 3:**
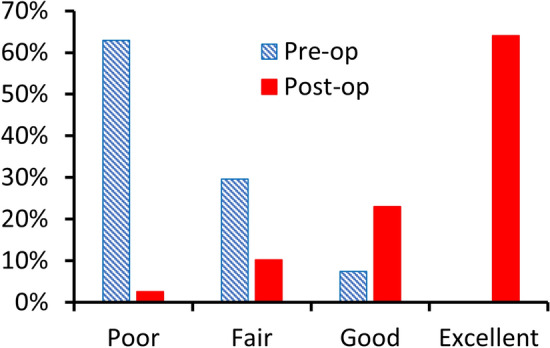
Categorical distribution of pre-operative Oxford Knee Score (OKS) and 1-year post-operative OKS according to Kalairajah et al. [10]

The mean OKS following FLO are presented in Table 3. At all time points, there was a statistically significant (*p < *0.0001) improvement in OKS compared to pre-operation. At 1 year the mean OKS was 41 with 87.2% of patients having an excellent (OKS > 41) or good score (34–41) [10] (Fig. 3). At 5 years the mean OKS was 40 (Table 3), however, at 5 years one patient had a poor OKS of 18. This patient reported various other comorbidities such as lower back pain and recovery from a broken neck that had severely affected their mobility and may have contributed to this low score. The mean TAS increased post-operatively, but the increase was not significant (*p = *0.51) (Table 3).

**Table 3:** Oxford knee score (OKS) and Tegner score for knees following conversion to bi-UKR with fixed lateral Oxford unicompartmental knee replacement

	Pre-bi-UKR operation	1-year post-operation	2-year post-operation	5-year post-operation	7-year post-operation
OKS mean (SD) [*n*]	21.5 (8.9) [*n = *27]	41.8 (5.9) [*n = *42]	42.2 (5.1) [*n = *37]	39.5 (8.8) [*n = *24]	45.0 (2.6) [*n = *4]
Tegner median (range) [*n*]	1.5 (0–8) [*n = *16]	3 (1–5) [*n = *41]	2.5 (1–6) [*n = *34]	2 (1–6) [*n = *20]	3 (1–3) [*n = *3]

**Fig. 4:**
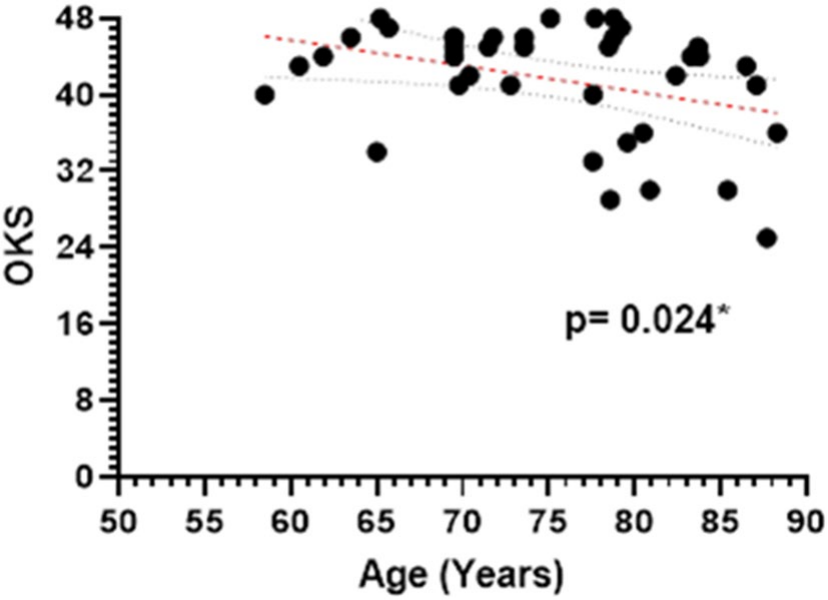
1-year post-operative Oxford Knee Score (OKS) versus patient age at the time of lateral UKR (*p = *0.02). Simple linear regression with 95% confidence bands shown

There were no statistically significant correlations between clinical outcome data and the extent of cartilage loss to the involved femur or tibia, gender, BMI, or time between medial and lateral UKR operations. There was a statistically significant negative correlation between age at the time of conversion to bi-UKR and 1-year OKS (*p = *0.02, Fig. 4).

## Discussion

This study found that very good results can be achieved when the FLO is used to treat lateral compartment failure after medial OUKR and appears to achieve clinical outcomes that are as good, if not better, than if a TKR was used. In this elderly patient cohort (mean age 74 at the time of FLO) there were no early complications, no re-operations related to the FLO and at 5 years there was a mean OKS of 40. Evidence from the literature would suggest that conversion to bi-UKR is advantageous over TKR, due to faster recovery, fewer complications, and better function, in appropriate circumstances [9, 12, 21]. The main concern with a staged bi-UKR is that similar to UKR, the procedure will have a higher failure rate than TKR in the long term [16]. However, as the progression of arthritis in the contralateral compartment can no longer occur, the reoperation rate of bi-UKR may well be comparable to that of TKR.

There was one re-operation 14 years after the initial medial OUKR, for a bearing exchange following dislocation of the medial mobile bearing. It is now recommended that the medial bearing thickness should be assessed on a well-aligned pre-operative AP radiograph, before the FLO. If the bearing appears very thin then, during the operation it should be replaced, and any associated impingement addressed. As increased wear is usually caused by impingement and impingement is the main cause of dislocation, this approach should prevent dislocation in the future.

At one and two years postoperatively, the mean OKS was 42, with 87% of the patients achieving an excellent or good OKS. These results are similar to those achieved following primary UKR [18]. A negative correlation between age and OKS at one year post-operatively (*p = *0.02) is to be expected as increasing age is associated with both decreasing activity and more major medical conditions [4]. There was an increase in mean TAS post-operatively, but this increase is not significant, as expected in an elderly population with low baseline activity. No correlations were found between clinical outcome scores and the severity of the damage to the lateral femoral or tibial condyle, gender, BMI, or time between medial and lateral UKR operations. This suggests that when considering whether to perform staged bi-UKR these factors need not be taken into consideration. However, the appropriate indications for the procedure need to be satisfied.

The literature would suggest that the early post-operative OKS of 42 for these patients, with lateral osteoarthritis following medial UKR, treated with lateral UKR are appreciably better than scores of patients, with lateral osteoarthritis following medial UKR, treated with TKR. For example, following conversion of UKR to TKR, Pearse et al. reported a mean OKS of 30 and Jonas et al. reported a mean OKS of 32 [9, 21]. However, the outcome of revisions of UKR to TKR is influenced by the reason for revision. As a conversion of a medial UKR with lateral osteoarthritis to TKR is usually a simple primary TKR the results may be better than those for conversions of UKR to TKR for other reasons. Kerens et al. reported a mean OKS at 1 year of 38 in a cohort that of patients mainly revised for lateral osteoarthritis, which is still not as good as the results of revision with a FLO. [12]. When comparing the difference in outcome of a medial UKR treated with lateral UKR or TKR, the most similar reports are those comparing bi-UKR and TKR. These studies have shown better results for bi-UKR in multiple scores including Western Ontario and McMaster Universities (WOMAC), Knee Society (KSS), OKS and EQ-5D scores [11, 14]. In addition, bi-UKR have improved biomechanical and functional results measured both in-vitro, using cadaveric knees, and in-vivo, using gate analysis [5]. These functional improvements were attributed to the bone and ACL-preserving nature of bi-UKR when compared to TKR.

In this study, the FLO were all implanted without medical complications, re-admissions, or early mortality which is to be expected considering the minimally invasive nature of the procedure. These results are supported by previous studies, showing a shorter recovery time for staged bi-UKR than TKR [8]. Furthermore, as the procedure is a minimally invasive UKR, the risk of medical complications such as stroke, myocardial infarction, thromboembolism, deep infection and early mortality is about half that of TKR [13]. This is particularly important as most patients undergoing these procedures are elderly. From the patient’s perspective, by the time they develop lateral osteoarthritis they have had a well-functioning medial UKR for many years. As a result, they tend not want their medial UKR removed and prefer a lateral UKR. Unlike national registries, they do not consider their medial UKR a failure and are pleased to have the opportunity to have another UKR.

The main limitation of the study is that the sample size is small, making it difficult to extrapolate conclusions to a larger population. However, lateral osteoarthritis after UKR, despite being the most common cause for revision, is rare so it is difficult to do a large study. Another limitation is that there was no matched group of patients with lateral osteoarthritis following medial UKR treated with TKR, so to compare outcomes we were reliant on the literature.

## Conclusion

The Fixed Lateral Oxford UKR seems to be a good treatment, at least for 5 years, for patients who have developed lateral osteoarthritis with a well-functioning medial UKR. The speed of recovery, incidence of medical complications, and functional outcome is similar to that reported for primary UKR and likely to be better than that achieved by a conversion to a TKR. However, longer follow-up is needed to draw firm conclusions.

## Data Availability

The participants of this study did not give written consent for their data to be shared publicly, so supporting data is not available.

## Abbreviations

Bi-UKR: Bi-unicompartmental knee replacement
DLO: Domed lateral Oxford unicompartmental knee replacement
FLO: Fixed lateral Oxford unicompartmental knee replacement
OKS: Oxford knee score
OUKR: Oxford unicompartmental knee replacement
TKR: Total knee replacement
